# Preoperative carbohydrate load and intraoperatively infused omega-3 polyunsaturated fatty acids positively impact nosocomial morbidity after coronary artery bypass grafting: a double-blind controlled randomized trial

**DOI:** 10.1186/s12937-017-0245-6

**Published:** 2017-04-20

**Authors:** Gibran Roder Feguri, Paulo Ruiz Lúcio de Lima, Danilo de Cerqueira Borges, Laura Ramos Toledo, Larissa Nadaf Batista, Thaís Carvalho e Silva, Neuber José Segri, José Eduardo de Aguilar-Nascimento

**Affiliations:** 1 Federal University of Mato Grosso, Cuiabá, Brazil; 2 General University Hospital, Cuiabá, Brazil; 3 UNIC - University of Cuiabá, Cuiabá, Brazil; 4 Cardiovascular Department, General University Hospital, Cuiabá, Brazil; 5 Nutrition Service, General University Hospital, Cuiaba, Brazil; 6 Department of Statistics, Federal University of Mato Grosso, Cuiabá, Brazil; 7 Federal University of Mato Grosso and UNIVAG Medical School, Varzea Grande, Brazil; 8 Rua Mal. Floriano Peixoto, 1520/503 – Duque de Caxias II, CEP:78045-395 Cuiabá, Brazil

**Keywords:** Myocardial revascularization, CAGB, Perioperative care, POAF, Fasting, Metabolism

## Abstract

**Background:**

A strategy of limited preoperative fasting, with carbohydrate (CHO) loading and intraoperative infusion of omega-3 polyunsaturated fatty acids (ω-3 PUFA), has seldom been tried in cardiovascular surgery. Brief fasting, followed by CHO intake 2 h before anesthesia, may improve recovery from CABG procedures and lower perioperative vasoactive drug requirements. Infusion of ω-3 PUFA may reduce occurrences of postoperative atrial fibrillation (POAF) and shorten hospital stays. The aim of this study was to assess morbidity (especially POAF) in ICU patients after coronary artery bypass grafting (CABG)/cardiopulmonary bypass (CPB) in combination, if preoperative fasts are curtailed in favor of CHO loading, and ω-3 PUFA are infused intraoperatively.

**Methods:**

Fifty-seven patients undergoing CABG were randomly assigned to receive 12.5% maltodextrin (200 ml, 2 h before anesthesia), without infusing ω-3 PUFA (CHO, *n* = 14); water (200 ml, 2 h before anesthesia), without infusing ω-3 PUFA (controls, *n* = 14); 12.5% maltodextrin (200 ml, 2 h before anesthesia) plus intraoperative ω-3 PUFA (0.2 mcg/kg) (CHO + W3, *n* = 15); or water (200 ml, 2 h before anesthesia) plus intraoperative ω-3 PUFA (0.2 mcg/kg) (W3, *n* = 14). Perioperative clinical variables and mortality were analyzed, examining the incidence of POAF, as well as the need for inotropic vasoactive drugs during surgery and in ICU.

**Results:**

Two deaths occurred (3.5%), but there were no instances of bronchoaspiration and mediastinitis. Neither ICU stays nor total postoperative stays differed by group (*P* > 0.05). Patients given preoperative CHO loads (CHO and CHO + W3 groups) experienced fewer instances of hospital infection (RR = 0.29, 95%CI 0.09–0.94; *P* = 0.023) and were less reliant on vasoactive amines during surgery (RR = 0.60, 95% CI 0.38–0.94; *P* = 0.020). Similarly, the number of patients requiring vasoactive drugs while recovering in ICU differed significantly by group (*P* = 0.008), showing benefits in patients given CHO loads. The overall incidence of POAF was 29.8% (17/57), differing significantly by group (*P* = 0.009). Groups given ω-3 PUFA (W3 and CHO + W3 groups) experienced significantly fewer instances of POAF (RR = 4.83, 95% CI 1.56–15.02; *P* = 0.001).

**Conclusion:**

Preoperative curtailment of fasting was safe in this cohort. When implemented in conjunction with CHO loading and infusion of ω-3 PUFA during surgery, expedited recovery from CABG with CPB was observed.

**Trial registration:**

NCT: 03017001

## Background

Omega-3 polyunsaturated fatty acids (ω-3 PUFA) are likely involved in proper balance of host immunity postoperatively. Although most studies of nutrients implicated in immunity pertain to abdominal surgery, there is some evidence that ω-3 PUFA is beneficial for patients undergoing cardiac surgery as well [1]. A recent meta-analysis has shown that ω-3 PUFA may reduce the incidence of postoperative atrial fibrillation (POAF) and shorten hospital stays [2, 3].

POAF is among the most common complications after coronary artery bypass grafting (CABG), ranging from 10 to 40% in incidence. Such events are known to impact healthcare costs, morbidity and mortality, postoperative discomfort, and tachycardia, necessitating rhythm reversal and prolonging ICU stays or forcing readmission. Hence, perioperative strategies to minimize occurrences of POAF are of great importance and are constantly under study [4, 5].

On the other hand, an abbreviated period of preoperative fasting with oral intake of carbohydrate (CHO) may offset insulin resistance after surgery and help reduce postoperative nausea and vomiting (PONV) [6]. In cardiac surgery specifically, some randomized trials have shown that CHO loading 2 h before inducing anesthesia will reduce ICU and hospital stays while reducing the need for perioperative vasoactive drug delivery [7, 8].

In our search of the current literature, no studies to date have combined preoperative CHO loading and intraoperative infusion of ω-3 PUFA in cardiac surgery. Presuming that this combination might confer some benefit, this study was conducted to investigate the effects of these two nutrients on morbidity in ICU (POAF primarily) suffered by patients who undergo CABG with cardiopulmonary bypass (CPB).

## Methods

This was a double-blind, controlled and randomized trial, examining patients subjected to CABG with CPB at General University Hospital, Cuiabá, Brazil between March, 2014 and June, 2016. The hospital’s Ethics Committee granted approval in advance (Protocol No 30493514.5.000.5165; 2014), and a Clinical Trials registration (NCT: 03017001) was obtained.

Patients of both genders (age range, 18–80 years) were included, each medically diagnosed with chronic coronary heart disease and eligible for elective CABG. All patients granted written informed consent. Grounds for exclusion were insulin-dependent diabetes, hepatic or renal disorders, thrombocytopenia, critical dyslipidemia (triglycerides 3-fold greater than established normal), gastroesophageal reflux, acute coronary syndromes, allergy to fish oil, and severe malnutrition. Candidates undergoing off-pump CABG, combined heart procedures, reoperations, or blood transfusion within 3 months prior were also excluded.

Using standard software (QuickCalcs [online]; GraphPad Software Inc, San Diego, CA, USA), subjects were randomly assigned to one of four groups as follows: 1) CHO (8-h fast for solids; 2-h fast plus 200-mL oral intake of 12.5% maltodextrin [25 g] in water; no intraoperative ω-3 PUFA); 2) controls (8-fast for solids; 2-h fast plus 200-mL oral intake of water only; no intraoperative ω-3 PUFA); 3) CHO + W3 (8-h fast for solids; 2-h fast plus 200-mL oral intake of 12.5% maltodextrin [25 g] in water; intraoperative infusion of ω-3 PUFA [0.2 mcg/kg over 4 h]); and 4) W3 (8-fast for solids; 2-h fast plus 200-mL oral intake of water only; intraoperative infusion of ω-3 PUFA [0.2 mcg/kg over 4 h]).

A hospital dietitian, as sole attendant privy to charted randomization, directed preoperative intake. Drinks delivered by ward nurse were given to patients before transport to the operative suite. The anesthesiologist was also informed (by dietician) of which patients would receive intraoperative ω-3 PUFA. None of the surgical team (surgeon or assistants) was aware of patient assignments. A team of cardiologists and intensive care physicians, also blind to study design and patient randomization, collected all data.

### Endpoints

Primary study endpoints were incidence of POAF and need of inotropic vasoactive drugs (dobutamine and/or noradrenalin) for weaning from CPB intraoperatively or postoperatively in ICU. As secondary endpoints, perioperative morbidity, hospital mortality, and durations of ICU and total postoperative hospitalization were analyzed.

### Anesthesia and surgical technique

The same surgeon (GRF) performed all procedures. Routine anesthetic techniques were used, namely induction via infusion of etomidate (0.2 mg/kg), fentanyl citrate (5 mcg/kg), and pancuronium bromide (0.1 mg/kg). Isoflurane was administered by inhalation route in usual doses (0.6-1.8%) until end of procedure for balanced general anesthesia. As maintenance, another bolus of fentanyl (2 mcg/kg), midazolam (0.1 mg/kg), and pancuronium bromide (0.05 mg/kg) was given, serving for muscle relaxation. All patients were kept under mechanical ventilation, with oxygen (FiO2) at 60% (or higher if needed).

Surgical entry was through median sternotomy, with CPB in the allotted time. The oxygenator used was membrane type (Braile Biomédica, São José do Rio Preto, Brazil). To protect myocardium, we used hypothermic intermittent anterograde blood cardioplegia (every 15–20 min), along with mild systemic hypothermia (33°–35 °C). All patients received intravenous cefuroxime (1.5 g) 1 h before inducing anesthesia, giving another dose after CPB. Maintenance doses (750 mg) were then administered every 6 h for 48 h. A bolus of methyl prednisolone (7 mg/kg) was delivered intravenously at anesthesia induction.

### Statistical analysis

The calculation of the sample size was based on the premise that the intervention with W3 + CHO protocol would reduce POAF by 50% versus controls. Assuming a β error (type II) of 20% and a α error (type I) of 5%, sample size calculations indicated 15 patients per group would be sufficient for this study. For values with Gaussian distributions, we used parametric paired (or non-paired) t-tests and analysis of variance for repeated measures, applying nonparametric tests (Friedman, Wilcoxon, and Mann-Whitney) to values with non-Gaussian distributions. Qualitative variables were assessed via chi-square and Fisher’s exact tests. Descriptive analyses were undertaken in Microsoft Office Excel 2007 (Microsoft Corp, Redmond, WA, USA), and statistical computations relied on SPSS v17.0 (SPSS Inc, Chicago, IL, USA). All testing was two-tailed at 80% power, with significance set at *P* < 0.05.

## Results

**Fig. 1:**
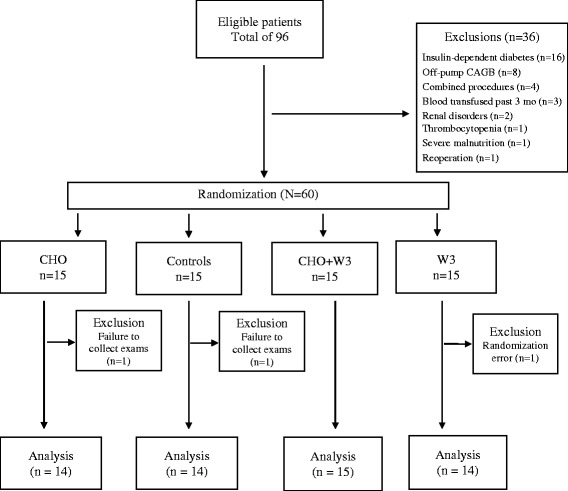
Flowchart of study design

A study flowchart is depicted in Fig. 1. Although 96 patients were eligible, 36 were excluded for various reasons. Ultimately, 60 were randomly assigned to the four groups; and after excluding one patient each from groups CHO, W3, and controls, 57 remained for analysis (CHO + W3: *n* = 15; others: *n* = 14, each). Nutritional and demographic data are shown by group in Table 1. In Table 2, clinical data of all groups are presented. All comparisons of the above showed these groups to be homogeneous.

**Table 1:** Demographic, nutritional, and clinical data of patient population (*N* = 57) by group

	CHO	Control	CHO + W3	W3	*P* value
	(*n* = 14)	(*n* = 14)	(*n* = 15)	(*n* = 14)	
Age (y/o)	60.86 ± 10.55 (63.00)	63.43 ± 8.56 (63.50)	59.93 ± 11.77 (63.00)	62.71 ± 10.90 (65.00)	0.797^a^
Males	12 (86)	10 (71)	7 (47)	9 (64)	0.160^c^
Caucasians	7 (50)	9 (64)	8 (53)	5 (36)	0.380^c^
Body weight (kg)	72.71 ± 12.58 (71,90)	72.82 ± 11.21 (71.20)	65.95 ± 10.69 (66.00)	73.78 ± 18.66 (69.20)	0.513^b^
Height (m)	1.67 ± 0.10 (1.64)	1.63 ± 0.08 (1.62)	1.64 ± 0.07 (1.64)	1.64 ± 0.10 (1.64)	0.663^a^
BMI (kg/m^2^)	26.26 ± 3.59 (26.60)	26.49 ± 3.38 (26.30)	25.27 ± 3.48 (25.40)	27.26 ± 5.64 (25.70)	0.911^b^
SGA-A (n; %)	11 (79)	13 (93)	13 (87)	13 (93)	0.617^c^

Data expressed as mean ± SD, median (in parentheses) for continuous variables, or number (%) for categorical variables

*BMI* body mass index, *SGA* subjective global assessment

^a^One way-ANOVA

^b^Kruskal-Wallis test

^c^Chi-square test

**Table 2:** Clinical data and risk factors of patient population (*N* = 57) by group

	CHO	Control	CHO + W3	W3	*P* value
	(*n* = 14)	(*n* = 14)	(*n* = 15)	(*n* = 14)	
LVEF	0.55 ± 0.12 (0.60)	0.57 ± 0.11 (0.60)	0.54 ± 0.13 (0.60)	0.62 ± 0.11 (0.65)	0.168^a^
Smoking	6 (43)	9 (64)	7 (47)	8 (57)	0.653^b^
Diabetes	4 (29)	3 (21)	5 (33)	6 (43)	0.666^b^
Dyslipidemia	11 (79)	12 (86)	8 (53)	11 (79)	0.204^b^
Previous AMI	7 (50)	9 (64)	6 (40)	7 (50)	0.631^b^
Beta-blocker use	9 (64)	10 (71)	6 (40)	11 (79)	0.151^b^
Statin use	11 (79)	11 (79)	9 (60)	11 (79)	0.580^b^
Fibrate use	2 (14)	2 (14)	2 (13)	3 (21)	0.930^b^
Previous PCI	1 (7)	4 (29)	4 (27)	3 (21)	0.495^b^

Data expressed as mean ± SD, median (in parentheses) for continuous variables, or number (%) for categorical variables

*LVEF* left ventricular ejection fraction, *AMI* acute myocardial infarction, *PCI* percutaneous coronary intervention

^a^Kruskal-Wallis test

^b^Chi-square test

### Intraoperative period

No patients suffered bronchial aspiration during intubation or after terminating anesthesia, and no intraoperative deaths occurred. Data from the intraoperative period are shown Table 3. There were no significant differences among the four groups.

**Table 3:** Intraoperative patient data (*N* = 57) by group

	CHO	Control	CHO + W3	W3	*P* value
	(*n* = 14)	(*n* = 14)	(*n* = 15)	(*n* = 14)	
Operative time (min)	244.64 ± 40.31 (240.00)	232.31 ± 49.27 (225.00)	249.29 ± 24.01 (240.00)	253.93 ± 40.86 (270.00)	0.532^a^
Duration of CPB (min)	77.14 ± 20.37 (82.50)	66.64 ± 24.50 (60.00)	63.53 ± 15.63 (66.00)	78.79 ± 22.93 (85.00)	0.147^a^
Duration of AC (min)	63.71 ± 17.29 (66.50)	56.86 ± 28.21 (50.50)	54.20 ± 15.52 (56.00)	61.57 ± 18.58 (68.00)	0.586^a^
Blood components transfused	7 (50)	7 (50)	7 (47)	8 (57)	0.953^b^
Number of grafts	3.00 ± 0.96 (3.00)	2.36 ± 0.93 (2.00)	2.67 ± 0.82 (3.00)	2.86 ± 0.86 (3.00)	0.266^a^
Complications	1 (7)	2 (14)	0 (0)	1 (7)	0.519^b^

Data expressed as mean ± SD, median (in parentheses) for continuous variables, or number (%) for categorical variables

*CPB* cardiopulmonary bypass, *AC* aortic clamping

^a^One-way ANOVA

^b^Chi-square test

The number of patients needing vasoactive drugs (dobutamine and/or noradrenaline) for weaning from CPB did not differ significantly by group (CHO: 7/14, 50.0%; controls: 12/14, 85.7%; CHO + W3: 6/15, 40.0%; W3: 9/14, 64.3%), although a tendency for control subjects to require such agents was noted (*P* = 0.071). Relative to other group members (W3 and control groups), patients given preoperative CHO loads (CHO and CHO + W3 groups) were less apt to require vasoactive amines (RR = 0.60, 95% CI 0.38–0.94; *P* = 0.020).

### Postoperative period

Clinical data from the postoperative period are shown in Table 4. ICU stays did not differ significantly by group (*P* = 0.713). Group means (medians) ± SD were as follows: CHO, 2.86 (2) ± 1.88 days; controls, 3.43 (2) ± 3.16 days; CHO + W3, 2.80 (3) ± 0.94 days; and W3, 3.36 (3) ± 1.55 days. Likewise, total postoperative stays did not differ significantly by group (*P* = 0.998). Group mean ± SD were as follows: CHO, 8.42 ± 7.79 days; controls, 8.07 ± 4.5 days; CHO + W3, 7.00 ± 1.51 days; and W3, 7.50 ± 2.77 days.

**Table 4:** Clinical data during postoperative patient recovery (*N* = 57) by group

	CHO	Control	CHO + W3	W3	*P* value
	(*n* = 14)	(*n* = 14)	(*n* = 15)	(*n* = 14)	
PONV at ICU	3 (21)	4 (29)	2 (13)	5 (36)	0.541^b^
Duration of MV (hours)	10.92 ± 11.12 (8.25)	8.47 ± 2.62 (8.50)	8.96 ± 4.01 (8.67)	8.21 ± 3.57 (7.50)	0.959^a^
Blood loss by12 h PO	353.57 ± 355.43 (300.00)	204.64 ± 99.78 (200.00)	233.33 ± 134.52 (200.00)	278.57 ± 169.52 (225.00)	0.351^a^
Vasoactive drug needed in ICU	3 (21)	10 (71)	3 (20)	8 (57)	0.008^b^
Blood components needed in ICU	6 (43)	5 (36)	3 (20)	3 (21)	0.470^b^
EVA	0 (0)	2 (14)	1 (7)	0 (0)	0.272^b^
AMI	1 (7)	0 (0)	0 (0)	1 (7)	0.557^b^
POAF	6 (43)	8 (57)	1 (7)	2 (14)	0.009^b^
Infectious complications	3 (21)	5 (36)	0 (0)	5 (36)	0.069^b^

Data expressed as mean ± SD, median (in parentheses) for continuous variable, or number (%) for categorical variables

*PONV* postoperative nausea and vomiting, *MV* mechanical ventilation, *PO* postoperative, *EVA* encephalic vascular accident, *POAF* postoperative atrial fibrillation, *AMI* acute myocardial infarction

^a^Kruskal-Wallis test

^b^Chi-square test

The number of patients needing vasoactive drugs (dobutamine and/or noradrenaline) during recovery in ICU did differ significantly by group (CHO, 3/14; 21.4%; controls, 10/14; 71.4%; CHO + W3, 3/15; 20.0%; and W3, 8/14; 57.1%) (*P* = 0.008). Notable differences were evident in both groups given preoperative CHO loads, compared with controls. No other calculations reached significance.

The overall incidence of POAF was 29.8% (17/57). However, rates differed significantly by group (*P* = 0.009). Those given ω-3 PUFA (W3 and CHO + W3 groups) experienced significantly less POAF than controls, and patients given both nutrients (CHO + W3) fared significantly better than those given CHO only (*P* = 0.035). Members of the CHO group did not differ significantly from controls (*P* = 0.571). Only 3 patients (10.3%) in the W3 and CHO + W3 groups developed POAF, compared with 14 (50.0%) in the other two groups (RR = 4.83, 95% CI 1.56–15.02; *P* = 0.001).

### Infectious complications and hospital mortality

There were no instances of postoperative mediastinitis. Thirteen patients (22.8%) experienced postoperative infections, specifically pneumonia (*n* = 9; two with sepsis) and wound site infections (*n* = 5). Although differences among groups approached significance (*P* = 0.069), as seen in Table 4, groups given preoperative CHO loads (CHO and CHO + W3 groups) incurred significantly fewer infections than the others (RR = 0.29, 95% CI 0.09–0.94; *P* = 0.023).

Overall, two deaths (3.5%) were recorded, one control subject and a member of the W3 group (*P* = 0.543). These occurred during ICU recovery. The control subject was a woman diagnosed with a stroke in the immediate postoperative period. She progressed to acute renal failure (requiring hemodialysis), sepsis, and then cardiogenic and septic shock. The W3 group member was a man diagnosed with perioperative acute myocardial infarction that led to refractory cardiogenic shock and acute renal failure.

## Discussion

In aggregate, these study outcomes indicate that administering preoperative CHO loads and intraoperative ω-3 PUFA is beneficial in the aftermath of CABG. Not only did fewer patients require vasoactive drugs for weaning from CPB or during ICU recovery, but also instances of POAF were significantly reduced. Apparently, sparing of vasoactive drugs is a function of oral CHO intake, whereas reduced rates of POAF are attributable to intraoperative infusion of ω-3 PUFA. In addition, groups given CHO-enriched drinks experienced fewer postoperative infections.

These results are in agreement with two previous studies that document speedy recovery and less need of vasoactive drugs, if oral CHO loading is done prior to cardiac surgery [7, 8]. Curtailing of preoperative fasting, with CHO loading, is no longer a novelty approach [9]. This practice of abandoning prolonged fasts is safe and has been endorsed by anesthesiology societies around the world [10, 11]. CHO-enriched drinks may ameliorate postoperative insulin sensitivity, decrease episodes of PONV, and alleviate preoperative sensations of thirst and hunger [6]. In cardiac surgery, there is not much data on the benefits of this particular strategy, so the present findings are especially relevant. As anticipated, there were no instances of bronchial aspiration in the two groups given CHO loads, nor in the groups receiving only water.

The benefits of ω-3 PUFA have been reported for decades in several areas of medicine, but primarily in the realms of cardiology and intensive care. Intravenous ω-3 PUFA is rapidly incorporated by cell membranes [12] and may reduce the production of pro-inflammatory mediators (eg, prostaglandins, thromboxanes, leukotrienes, platelet-activating factor, and cytokines) [13, 14]. Such effects have been credited for reduced hospital stays [15] and lower rates of severe infection [16] in critically ill patients, fewer postoperative complications [17], and possibly lower mortality in acute lung injury [18]. In the field of cardiology, other benefits include declines in morbidity and mortality from congestive heart failure and in sudden death from infarction [19]. The administering of ω-3 PUFA is also linked with better control of dyslipidemia, heart rate, and chronic atrial fibrillation [20, 21]. However, data in the literature are controversial, some studies failing to confirm these benefits [22].

A number of investigations have addressed the benefits of perioperative ω-3 PUFA infusion, or even preoperative oral treatment, in the context of cardiovascular surgery. In a study by Berger et al. [1], two randomly assigned groups were compared, one a placebo (control) group. The other was given infusions of ω-3 PUFA (0.2 g/kg) 12 h and 2 h preoperatively and immediately following surgery. A significant decline in postoperative IL-6 (cytokine) was found in the ω-3 PUFA group, as well as a reduced incidence of arrhythmias, but without significant between-group differences. Our study showed a lower incidence of POAF, as reported in a meta-analysis by Langlois et al. [3]. Their efforts encompassed a systematic review of 19 randomized clinical trials, for a total of 4335 patients undergoing cardiac surgery. Similar to our study, this meta-analysis found no effect of ω-3 PUFA on duration of ICU stays and postoperative mortality rate. However, this meta-analysis showed that infusion of ω-3 PUFA was associated with shorter hospital stays and lower incidence of POAF, especially with use of CPB. The reductions of POAF seen in our patients and in the meta-analysis above perhaps reflect a dampening of inflammatory response in this setting, induced by intraoperative infusion of ω-3 PUFA.

Outcomes of the present study must be interpreted with caution, because of the small number of patients included. Indeed, further studies on this issue are warranted. Nonetheless, we may conclude that perioperative use of these two nutrients seems beneficial for recovery of patients undergoing CABG.

## Abbreviations

CABG: Coronary artery bypass grafting
CHO: Carbohydrate
CPB: Cardiopulmonary bypass
ICU: Intensive care unit
POAF: Postoperative atrial fibrillation
PONV: Postoperative nausea and vomiting
ω-3 PUFA: Omega-3 polyunsaturated fatty acids
